# Supplementary material for: Mechanisms governing the pioneering and redistribution capabilities of the non-classical pioneer PU.1
Source: Nat Commun. 2020 Jan 21;11:402. doi: 10.1038/s41467-019-13960-2 (PMC6972792; doi:10.1038/s41467-019-13960-2)
Supplement: Supplementary file 7 — Source data [file 41467_2019_13960_MOESM7_ESM.zip › Source_Data/FigureS3/FigureS3H_MotifScanOutput/homerResults/motif23.similar.html]

motif23

## Information for motif23

C
A
T
G
C
A
T
G
G
A
T
C
G
A
C
T
A
G
C
T
C
G
A
T
A
T
G
C
G
C
T
A
T
A
G
C
C
G
T
A
A
T
C
G
G
T
C
A
  
Reverse Opposite:  

C
A
G
T
A
T
G
C
G
C
A
T
A
T
C
G
C
G
A
T
T
A
C
G
G
C
T
A
T
C
G
A
C
T
G
A
C
T
A
G
G
T
A
C
G
T
A
C
  

|  |  |
| --- | --- |
| p-value: | 1e-37 |
| log p-value: | -8.566e+01 |
| Information Content per bp: | 1.588 |
| Number of Target Sequences with motif | 766.0 |
| Percentage of Target Sequences with motif | 19.81% |
| Number of Background Sequences with motif | 5460.0 |
| Percentage of Background Sequences with motif | 12.19% |
| Average Position of motif in Targets | 308.0 +/- 316.5bp |
| Average Position of motif in Background | 210.2 +/- 143.9bp |
| Strand Bias (log2 ratio + to - strand density) | -0.1 |
| Multiplicity (# of sites on avg that occur together) | 1.11 |
| Motif File: | file (matrix) reverse opposite |

### Similar de novo motifs found

|  |  |  |  |  |  |  |  |
| --- | --- | --- | --- | --- | --- | --- | --- |
| Rank | Match Score | Redundant Motif | P-value | log P-value | % of Targets | % of Background | Motif file |
| 1 | 0.796 | A T C G A T G C G A C T G A C T C A G T A G T C G C T A T C G A C T G A A T C G G T A C | 1e-25 | -58.014757 | 9.70% | 5.29% | motif file (matrix) |
| 2 | 0.764 | G A C T T A G C C G A T A T C G C G T A T C A G C G T A T C A G C G T A C T A G A G T C | 1e-19 | -45.276449 | 10.86% | 6.67% | motif file (matrix) |
